# Five-year results of heart rate control with ivabradine or metoprolol succinate in patients after heart transplantation

**DOI:** 10.1007/s00392-020-01692-z

**Published:** 2020-06-22

**Authors:** Rasmus Rivinius, Matthias Helmschrott, Ann-Kathrin Rahm, Fabrice F. Darche, Dierk Thomas, Tom Bruckner, Andreas O. Doesch, Hugo A. Katus, Philipp Ehlermann

**Affiliations:** 1 Department of Cardiology, Angiology and Pneumology, Heidelberg University Hospital, Im Neuenheimer Feld 410, 69120 Heidelberg, Germany; 2 Heidelberg Center for Heart Rhythm Disorders (HCR), Heidelberg University Hospital, Heidelberg, Germany; 3 German Center for Cardiovascular Research (DZHK), Partner Site Heidelberg/Mannheim, Heidelberg, Germany; 4 Institute for Medical Biometry and Informatics, University of Heidelberg, Heidelberg, Germany; 5 Department of Pneumology and Oncology, Asklepios Hospital, Bad Salzungen, Germany

**Keywords:** Heart rate, Heart transplantation, Ivabradine, Metoprolol succinate, Mortality

## Abstract

**Background:**

Cardiac graft denervation causes inadequate sinus tachycardia in patients after heart transplantation (HTX) which is associated with reduced survival. This study investigated the 5-year results of heart rate control with ivabradine or metoprolol succinate in patients after HTX.

**Methods:**

This registry study analyzed 104 patients receiving either ivabradine (*n* = 50) or metoprolol succinate (*n* = 54) within 5 years after HTX. Analysis included patient characteristics, medication, echocardiographic features, cardiac catheterization data, cardiac biomarkers, heart rates, and post-transplant survival including causes of death.

**Results:**

Demographics and post-transplant medication revealed no significant differences except for ivabradine and metoprolol succinate use. At 5-year follow-up, patients with ivabradine had a significantly lower heart rate (73.3 bpm) compared to baseline (88.6 bpm; *P* < 0.01) and to metoprolol succinate (80.4 bpm; *P* < 0.01), a reduced left ventricular mass (154.8 g) compared to baseline (179.5 g; *P* < 0.01) and to metoprolol succinate (177.3 g; *P* < 0.01), a lower left ventricular end-diastolic pressure (LVEDP; 12.0 mmHg) compared to baseline (15.5 mmHg; *P* < 0.01) and to metoprolol succinate (17.1 mmHg; *P* < 0.01), and a reduced NT-proBNP level (525.4 pg/ml) compared to baseline (3826.3 pg/ml; *P* < 0.01) and to metoprolol succinate (1038.9 pg/ml; *P* < 0.01). Five-year post-transplant survival was significantly better in patients with ivabradine (90.0%) versus metoprolol succinate (68.5%; *P* < 0.01).

**Conclusion:**

Patients receiving ivabradine showed a superior heart rate reduction and a better left ventricular diastolic function along with an improved 5-year survival after HTX.

## Introduction

Elevated resting heart rates have been associated with increased morbidity and mortality in patients with heart failure as well as in the general population without known heart disease [1–8]. Patients after heart transplantation (HTX) often suffer from sinus tachycardia as a result of cardiac denervation [9–14]. Inadequately elevated resting heart rates cause increased myocardial oxygen demand, a decreased stroke volume by shortened diastolic filling, and a reduced myocardial perfusion [12–14]. Higher resting heart rates have consequently been related to higher mortality in patients after HTX [14, 15].

Regulation of resting heart rate in patients after HTX is limited to humoral factors due to suspended autonomous cardiac control, although partial post-transplant cardiac re-innervation has been reported [9–17]. To achieve physiological resting heart rates and to avoid inappropriate sinus tachycardia in patients after HTX, pharmacological heart rate reduction is a suitable option. A specific and selective drug with a minimum of side-effects is preferable for this purpose [9–14]. However, common heart rate reduction medication comprises beta blockers and non-dihydropyridine calcium channel blockers which are non-specific inhibitors of pacemaker activity [9–14, 18, 19] Both drug classes have cardiac and systemic side-effects such as atrioventricular block, hypotension, negative inotropy, bronchospasm, depression, fatigue, and sexual dysfunction [9–14, 20].

A specific and selective inhibitor of pacemaker activity is ivabradine which inhibits the so-called funny current (*I*_f_), also known as “pacemaker current”, generated by hyperpolarization-activated cyclic nucleotide-gated (HCN) channels in pacemaker cells [20–24]. Inhibition of *I*_f_ causes a prolongation of the spontaneous diastolic depolarization in sinoatrial node cells reducing resting heart rate without negative effects on atrioventricular conduction, blood pressure or inotropy [9–14, 20]. In addition to heart rate reduction, the use of ivabradine has been associated with improved left ventricular (LV) function, improved myocardial structure, and reduced LV filling pressure [25–28].

Former studies focused on short and mid-term effects of ivabradine in patients after HTX.[9–14] Information on long-term data of ivabradine in patients after HTX is not available. To fill the gap of evidence, this study was designed to investigate 5-year results of ivabradine or metoprolol succinate in patients after HTX on resting heart rate, LV mass, left ventricular ejection fraction (LVEF), LV diastolic function, cardiac catheterization data, cardiac biomarkers, and survival after HTX.

## Patients and methods

### Patients

We performed this study in accordance with the ethical principles for medical research of the Declaration of Helsinki. Approval was given by the institutional review board (IRB) of Heidelberg University (ethical approval number: S-286/2015). Written informed consent was obtained from patients for inclusion in the Heidelberg HTX Registry allowing the clinical and scientific use of data. According to the ethical approval, no additional written informed consent was required for this study as only routine clinical data were analyzed [14, 28–33].

This study included all adult patients (≥ 18 years) with continuous post-transplant use of ivabradine or metoprolol succinate (in the following context referred to as metoprolol) receiving HTX at Heidelberg Heart Center between 2006 and 2014. Patients were excluded if they were only temporarily treated with ivabradine or metoprolol, received a combination of ivabradine and metoprolol, or were treated with additional antiarrhythmic drugs (amiodarone, digoxin/digitoxin, other beta blocker, or non-dihydropyridine calcium channel blocker) [14].

Five-year follow-up data could be obtained from all 104 patients and no patient was lost to follow-up. As the use of ivabradine in patients after HTX is still off-label, [34] patients were explicitly informed about effects, adverse effects, contraindications, and the off-label use of ivabradine. We did not perform a preselection or randomization of patients after HTX regarding the application of ivabradine or metoprolol for heart rate reduction. Individual physician practice and patient preference influenced the prescription of either drug reflecting real-world data [14].

### Follow-up

Patients were continuously cared for by the medical team of the Heidelberg Heart Center. After hospital discharge, patients presented at the HTX outpatient-clinic for baseline follow-up. Patients were hereafter seen once a month until 6 months after HTX, then bimonthly between post-transplant month six to twelve, and subsequently four times per year. During follow-up, patients were routinely questioned about their medication intake, side effects, and problems related to medication in order to ensure proper adherence to medication. Routine follow-up included medical history taking, physical examination, 12-lead electrocardiogram (ECG), echocardiography, systolic and diastolic blood pressure measurement, and routine laboratory analysis including immunosuppressive drug monitoring [14, 28–33].

### Post-transplant medication

Post-transplant medication including immunosuppressive drug therapy was administered in accordance to center standard. Patients initially received an anti-thymocyte globulin-based immunosuppression induction therapy after HTX. The majority of patients received an immunosuppressive drug regimen consisting of tacrolimus (TAC) and mycophenolate mofetil (MMF) as the initial immunosuppressive regime of cyclosporine A (CsA) and mycophenolate mofetil (MMF) was subsequently replaced by tacrolimus (TAC) and MMF from 2006 onward. Additionally, patients received steroids (prednisolone) which were tapered incrementally and were finally discontinued (if clinically possible) 6 months after HTX [14, 28–33].

### Statistical analysis

Statistical analysis of data was performed with SAS software (Version 9.4, SAS Institute, Cary, NC, USA). Data were expressed as count (*n*) with percentage (%) or as mean ± standard deviation (SD). Mean difference (MD) or hazard ratio (HR) with 95% confidence interval (CI) were used as measures of association. Student's *t* test was applied for continuous variables and chi-squared test was used for categorical variables. Kaplan–Meier estimator was employed to graphically display 5-year post-transplant survival. A *P* value of < 0.05 was considered statistically significant [14, 28–33].

Large-scale univariate analyses were carried out to search for intergroup differences including recipient data, previous open-heart surgery, principal diagnosis for HTX, donor data, transplant sex mismatch, perioperative data, immunosuppressive drug therapy, post-transplant medication, echocardiographic features after HTX, cardiac catheterization data after HTX, and cardiac biomarkers after HTX. Course of heart rates [beats per minute (bpm)] in patients receiving ivabradine or metoprolol within 5 years after HTX were assessed by resting 12-lead ECG at baseline, 6, 12, 18, 24, 30, 36, 42, 48, 54, and 60-month follow-up after HTX. Moreover, 24-h Holter monitoring was used to determine average resting heart rate. Causes of death within 5 years after HTX were examined using the following categories: transplant failure, acute rejection, infection/sepsis, malignancy, and thromboembolic event/bleeding. Evaluation of 5-year post-transplant mortality between patients receiving ivabradine or metoprolol after HTX further included a multivariate analysis (Cox regression model) with the following five clinically relevant parameters based on a predetermined model: administration of ivabradine after HTX (in total), recipient age (years), donor age (years), transplant sex mismatch (in total), and ischemic time (min). We did not include additional parameters in this multivariate analysis to avoid biased regression coefficients and to ensure a stable number of events (deceased patients) per analyzed variable [14, 28–33].

## Results

### Demographics and medication after heart transplantation

A total of 246 adult patients (age ≥ 18 years) excluding cardiac re-transplantations received HTX at Heidelberg Heart Center between 2006 and 2014. After application of the above-mentioned selection criteria, 104 patients could be included in this 5-year analysis from the initial post-transplant baseline visit until 5-year post-transplant follow-up: 50 patients with ivabradine (48.1%) and 54 patients with metoprolol (51.9%).

In terms of demographics, the two groups showed no statistically significant differences in recipient data, previous open-heart surgery of the recipient, principal diagnosis for HTX, donor data, transplant sex mismatch, or perioperative data (all *P* ≥ 0.05). Demographic and clinical characteristics are provided in Table 1.

**Table 1:** Demographic and clinical characteristics at baseline

Parameter	Ivabradine	Metoprolol	Difference	95% CI	*P* value
	(*n* = 50)	(*n* = 54)			
Recipient data					
Age (years), mean ± SD	49.9 ± 11.2	52.6 ± 10.2	2.7 years	− 1.6 to 7.0 years	0.22
Male sex, *n* (%)	38 (76.0%)	40 (74.1%)	1.9%	− 14.7 to 18.5%	0.82
Body mass index (kg/m^2^), mean ± SD	24.8 ± 4.9	25.9 ± 4.4	1.1 kg/m^2^	− 0.7 to 2.9 kg/m^2^	0.23
Arterial hypertension, *n* (%)	26 (52.0%)	35 (64.8%)	12.8%	− 6.0 to 31.6%	0.18
Dyslipidemia, *n* (%)	29 (58.0%)	33 (61.1%)	3.1%	− 15.8 to 22.0%	0.75
Diabetes mellitus, *n* (%)	16 (32.0%)	20 (37.0%)	5.0%	− 13.2 to 23.2%	0.59
Renal insufficiency^a^, *n* (%)	24 (48.0%)	31 (57.4%)	9.4%	− 9.7 to 28.5%	0.34
GFR (ml/min/1.73 m^2^), mean ± SD	63.4 ± 24.3	57.7 ± 20.2	5.7 ml/min/1.73 m^2^	− 3.1 to 14.5 ml/min/1.73 m^2^	0.20
Previous open-heart surgery					
Overall open-heart surgery, *n* (%)	11 (22.0%)	10 (18.5%)	3.5%	− 12.0 to 19.0%	0.66
CABG surgery, *n* (%)	3 (6.0%)	5 (9.3%)	3.3%	− 6.9 to 13.5%	0.53
Congenital, valvular or ventricular surgery, *n* (%)	4 (8.0%)	4 (7.4%)	0.6%	− 9.7 to 10.9%	0.91
VAD surgery, *n* (%)	5 (10.0%)	3 (5.6%)	4.4%	− 5.9 to 14.7%	0.40
Principal diagnosis for HTX					
Ischemic CMP, *n* (%)	14 (28.0%)	22 (40.7%)	12.7%	− 5.3 to 30.7%	0.17
Non-ischemic CMP, *n* (%)	28 (56.0%)	22 (40.7%)	15.3%	− 3.7 to 34.3%	0.12
Valvular heart disease, *n* (%)	2 (4.0%)	1 (1.9%)	2.1%	− 4.4 to 8.6%	0.51
Cardiac amyloidosis, *n* (%)	6 (12.0%)	9 (16.7%)	4.7%	− 8.7 to 18.1%	0.50
Donor data					
Age (years), mean ± SD	42.0 ± 14.6	47.2 ± 12.1	5.2 years	− 0.2 to 10.6 years	0.06
Male sex, *n* (%)	12 (24.0%)	18 (33.3%)	9.3%	− 7.9 to 26.5%	0.29
Body mass index (kg/m^2^), mean ± SD	24.4 ± 4.5	26.1 ± 5.1	1.7 kg/m^2^	− 0.2 to 3.6 kg/m^2^	0.08
Transplant sex mismatch					
Mismatch, *n* (%)	31 (62.0%)	24 (44.5%)	17.5%	− 1.3 to 36.3%	0.07
Donor (m) to recipient (f), *n* (%)	2 (4.0%)	1 (1.9%)	2.1%	− 4.4 to 8.6%	0.51
Donor (f) to recipient (m), *n* (%)	29 (58.0%)	23 (42.6%)	15.4%	− 3.6 to 34.4%	0.12
Perioperative data					
Ischemic time (min), mean ± SD	272.3 ± 52.1	256.6 ± 59.9	15.7 min	− 6.3 to 37.7%	0.16
Biatrial HTX, *n* (%)	0 (0.0%)	1 (1.9%)	1.9%	− 1.7 to 5.5%	0.33
Bicaval HTX, *n* (%)	24 (48.0%)	19 (35.2%)	12.8%	− 6.0 to 31.6%	0.18
Total orthotopic HTX, *n* (%)	26 (52.0%)	34 (62.9%)	10.9%	− 8.0 to 29.8%	0.26
Length of initial hospital stay (days), mean ± SD	43.9 ± 19.7	46.3 ± 19.0	2.4 days	− 5.2 to 10.0%	0.54

*CABG* coronary artery bypass graft, *CMP* cardiomyopathy, *f* female, *GFR* glomerular filtration rate, *HTX* heart transplantation, *m* male, *n* = number, *SD* standard deviation, *VAD* ventricular assist device

^a^Glomerular filtration rate < 60 ml/min/1.73 m^2^

Further analysis of post-transplant medication including the immunosuppressive drug regimen revealed no statistically significant differences between both groups (all *P* ≥ 0.05) except for the administration of ivabradine or metoprolol. Post-transplant medication including immunosuppressive drug regimen is given in Table 2.

**Table 2:** Post-transplant medication at baseline

Parameter	Ivabradine	Metoprolol	Difference (%)	95% CI	*P *value
	(*n* = 50)	(*n* = 54)			
Cyclosporine A, *n* (%)	4 (8.0%)	5 (9.3%)	1.3	− 9.5 to 12.1%	0.82
Tacrolimus, *n* (%)	46 (92.0%)	49 (90.7%)	1.3	− 9.5 to 12.1%	0.82
Azathioprine, *n* (%)	0 (0.0%)	0 (0.0%)	0.0	n.a.	n.a.
Mycophenolate mofetil, *n* (%)	50 (100.0%)	54 (100.0%)	0.0	n.a.	n.a.
Steroids, *n* (%)	50 (100.0%)	54 (100.0%)	0.0	n.a.	n.a.
ASA, *n* (%)	7 (14.0%)	12 (22.2%)	8.2	− 6.5 to 22.9%	0.28
Amiodarone, *n* (%)	0 (0.0%)	0 (0.0%)	0.0	n.a.	n.a.
Digitalis, *n* (%)	0 (0.0%)	0 (0.0%)	0.0	n.a.	n.a.
Beta blocker, *n* (%)	0 (0.0%)	54 (100.0%)	100.0	n.a.	< 0.01*
Ivabradine, *n* (%)	50 (100.0%)	0 (0.0%)	100.0	n.a.	< 0.01*
Calcium channel blocker	11 (22.0%)	12 (22.2%)	0.2	− 15.8 to 16.2%	0.98
Dihydropyridine, *n* (%)	11 (22.0%)	12 (22.2%)	0.2	− 15.8 to 16.2%	0.98
Non-dihydropyridine, *n* (%)	0 (0.0%)	0 (0.0%)	0.0	n.a.	n.a.
ACE inhibitor/ARB, *n* (%)	22 (44.0%)	23 (42.6%)	1.4	− 17.7 to 20.5%	0.88
Diuretic, *n* (%)	50 (100.0%)	54 (100.0%)	0.0	n.a.	n.a.
Statin, *n* (%)	35 (70.0%)	41 (75.9%)	5.9	− 11.2 to 23.0%	0.50
Gastric protection (PPI/H_2_ blocker), *n* (%)	50 (100.0%)	54 (100.0%)	0.0	n.a.	n.a.

*ASA* acetylsalicylic acid, *ACE inhibitor* angiotensin-converting-enzyme inhibitor, *ARB* angiotensin II receptor blocker, *PPI* proton pump inhibitor, *H*_*2*_* blocker* histamine receptor blocker, *n* = number, *n.a.* not applicable

*Statistically significant (*P* < 0.05)

### Heart rates after heart transplantation

**Fig. 1:**
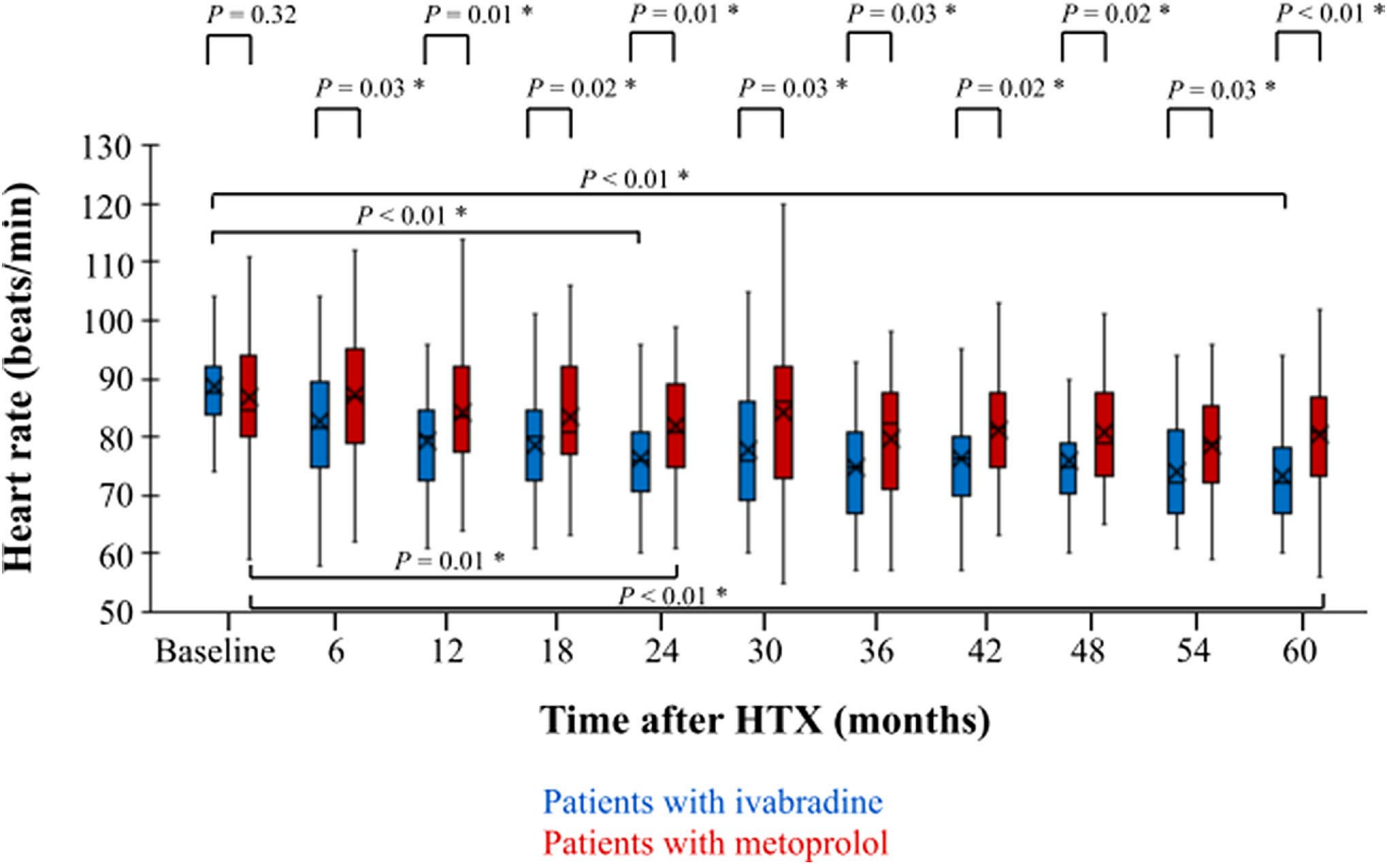
Course of heart rates in patients with ivabradine or metoprolol within 60 months after HTX. Baseline heart rate after HTX indicated no significant difference between patients with ivabradine vs. metoprolol (*P* = 0.32). At 24-month follow up after HTX, patients receiving ivabradine showed a statistically lower heart in comparison to baseline (*P* < 0.01) and to patients with metoprolol at 24-month follow-up (*P* = 0.01). At 60-month follow up after HTX, patients receiving ivabradine kept having a statistically lower heart in comparison to baseline (*P* < 0.01) and to patients with metoprolol at 60-month follow-up (*P* < 0.01). *HTX* heart transplantation, *statistically significant (*P* < 0.05)

Baseline resting heart rates were comparable between both groups (ivabradine group: 88.6 ± 7.8 bpm vs. metoprolol group: 86.9 ± 9.6 bpm; *P* = 0.32). After 2 years, patients with ivabradine had a statistically significant lower resting heart rate in comparison to the initial baseline visit (ivabradine 24-month follow-up: 76.2 ± 9.7 bpm vs. ivabradine baseline visit: 88.6 ± 7.8 bpm; *P* < 0.01) and to patients with metoprolol (ivabradine 24-month follow-up: 76.2 ± 9.7 bpm vs. metoprolol 24-month follow-up: 81.8 ± 10.3 bpm; *P* = 0.01). This difference in resting heart rate was still present at 5-year post-transplant follow-up as patients with ivabradine continued to have a significantly lower resting heart rate compared to the initial baseline visit (ivabradine 60-month follow-up: 73.3 ± 9.1 bpm vs. ivabradine baseline visit: 88.6 ± 7.8 bpm; *P* < 0.01) and to patients with metoprolol (ivabradine 60-month follow-up: 73.3 ± 9.1 bpm vs. metoprolol 60-month follow-up: 80.4 ± 10.1 bpm; *P* < 0.01). Course of resting heart rates within 60 months after HTX is shown in Fig. 1.

Analysis of 24-h Holter monitoring at baseline showed no significant intergroup difference in average heart rates (ivabradine group: 86.2 ± 10.0 bpm vs. metoprolol group: 85.8 ± 9.1 bpm; *P* = 0.82). At 2-year follow-up, Holter monitoring revealed a significantly lower average heart rate in the ivabradine group compared to the initial baseline visit (ivabradine 24-month follow-up: 76.6 ± 9.4 bpm vs. ivabradine baseline visit: 86.2 ± 10.0 bpm; *P* < 0.01) and to patients with metoprolol (ivabradine 24-month follow-up: 76.6 ± 9.4 bpm vs. metoprolol 24-month follow-up: 81.8 ± 8.4 bpm; *P* = 0.01). At 5-year follow-up, Holter monitoring still showed a significantly lower average heart rate in the ivabradine group compared to the initial baseline visit (ivabradine 60-month follow-up: 72.9 ± 6.9 bpm vs. ivabradine baseline visit: 86.2 ± 10.0 bpm; *P* < 0.01) and to patients with metoprolol (ivabradine 60-month follow-up: 72.9 ± 6.9 bpm vs. metoprolol 60-month follow-up: 79.5 ± 8.2 bpm; *P* < 0.01).

### Drug dosage of ivabradine and metoprolol after heart transplantation

At the initial post-transplant baseline visit, patients with ivabradine had a mean daily dose of 9.8 mg ± 3.0 mg ranging from 5.0 to 15.0 mg and patients with metoprolol had a mean daily dose of 97.2 mg ± 45.2 mg ranging from 47.5 to 190.0 mg. At 2-year post-transplant follow-up, mean daily ivabradine dose was 10.8 ± 3.6 mg ranging from 5.0 to 15.0 mg and mean daily metoprolol dose was 106.6 ± 47.9 mg ranging from 47.5 to 190.0 mg. At 5-year post-transplant follow-up, mean daily ivabradine dose was 10.5 mg ± 3.5 mg ranging from 5.0 to 15.0 mg and mean daily metoprolol dose was 116.1 mg ± 51.3 mg ranging from 47.5 to 190.0 mg.

### Blood pressure and side effects after heart transplantation

Assessment of blood pressure at baseline showed no significant differences between groups concerning systolic blood pressure (ivabradine group: 125.6 ± 14.0 mmHg vs. metoprolol group: 125.3 ± 17.0 mmHg; *P* = 0.92) or diastolic blood pressure (ivabradine group: 77.8 ± 9.3 mmHg vs. metoprolol group: 76.8 ± 9.0 mmHg; *P* = 0.57).

After 2 years, patients with ivabradine or metoprolol showed a comparable systolic blood pressure (ivabradine group: 125.3 ± 12.9 mmHg vs. metoprolol group: 125.1 ± 11.9 mmHg; *P* = 0.94) and diastolic blood pressure (ivabradine group: 76.9 ± 9.1 mmHg vs. metoprolol group: 77.7 ± 8.2 mmHg; *P* = 0.69). At 5-year post-transplant follow-up, groups showed no significant difference regarding systolic blood pressure (ivabradine group: 125.7 ± 13.2 mmHg vs. metoprolol group: 125.8 ± 9.5 mmHg; *P* = 0.96) or diastolic blood pressure (ivabradine group: 76.8 ± 7.5 mmHg vs. metoprolol group: 77.5 ± 8.6 mmHg; *P* = 0.69).

The use of ivabradine was generally well tolerated. Patients reported only a few side effects in the initial period including three patients (6.0%) with transient experiences of phosphenes. No patient (0.0%) in the ivabradine group had symptomatic bradycardia, whereas five patients (9.3%; *P* = 0.03) in the metoprolol group reported about symptomatic bradycardia with heart rates < 60 bpm. In addition, one patient (2.0%) in the ivabradine group had intermittent dizziness, while seven patients in the metoprolol group stated dizziness (13.0%; *P* = 0.04). No patient (0.0%) in the ivabradine group had fatigue, whereas five patients in the metoprolol group complained about fatigue (9.3%; *P* = 0.03). Six male patients of advanced age in the metoprolol group (11.1%) mentioned intermittent sexual dysfunction while no patient in the ivabradine group (0.0%; *P* = 0.02) reported about sexual dysfunction.

### Mortality and courses of death after heart transplantation

**Fig. 2:**
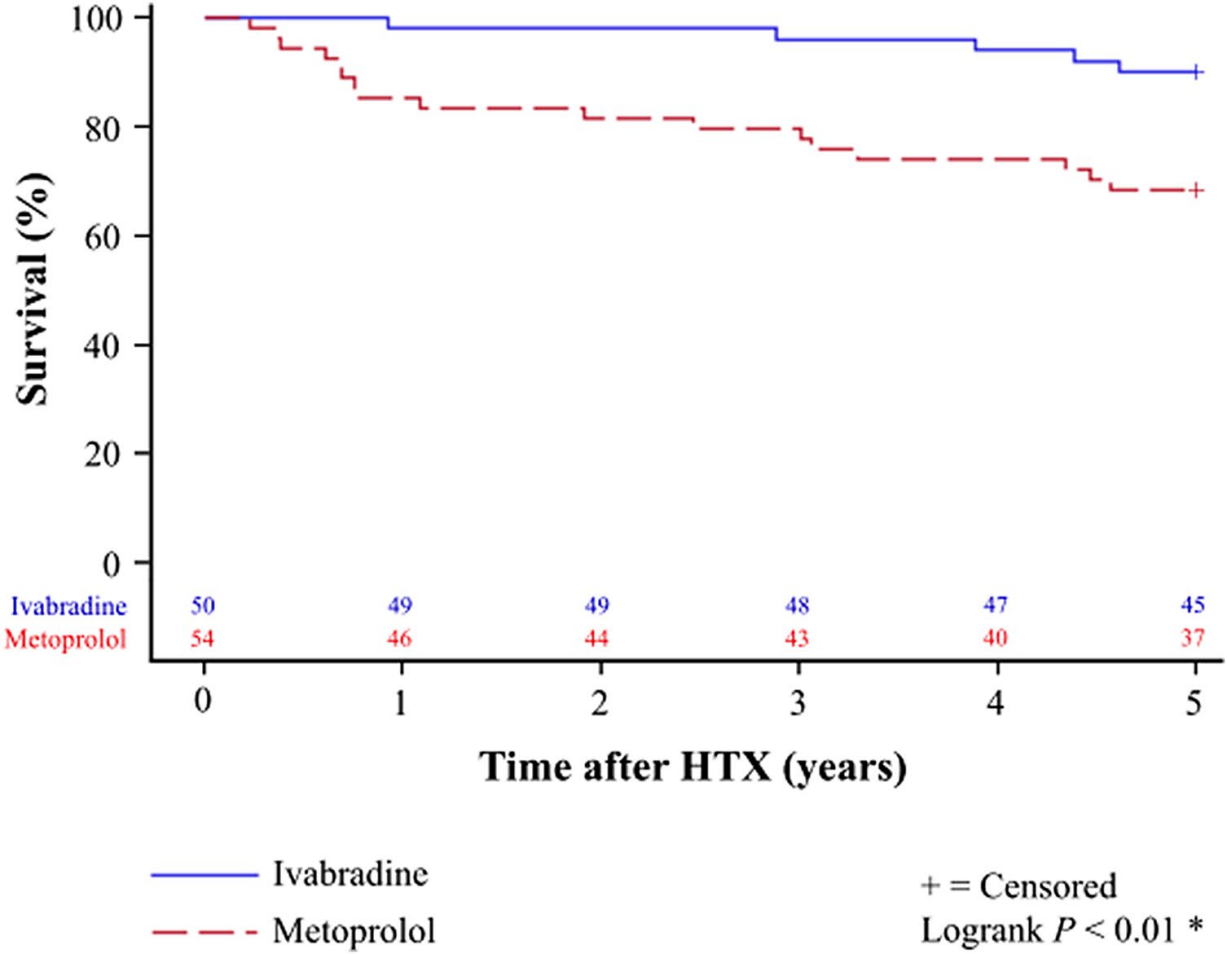
5-year survival after HTX in patients with ivabradine or metoprolol (Kaplan–Meier estimator). Patients with ivabradine showed a statistically significant superior 24-month [49 of 50 patients (98.0%) vs. 44 of 54 patients (81.5%); *P* < 0.01] and 60-month [45 of 50 patients (90.0%) vs. 37 of 54 patients (68.5%); *P* < 0.01)] post-transplant survival in comparison to patients with metoprolol. *HTX* heart transplantation, *statistically significant (*P* < 0.05)

Kaplan–Meier analysis showed a significantly better 24-month survival [ivabradine group: 49 of 50 patients (98.0%) vs. metoprolol group: 44 of 54 patients (81.5%); *P* < 0.01] as well as a better 60-month survival [ivabradine group: 45 of 50 patients (90.0%) vs. metoprolol group: 37 of 54 patients (68.5%); *P* < 0.01] in patients with ivabradine after HTX. Kaplan–Meier analysis for 5-year post-transplant survival is provided in Fig. 2.

Regarding the causes of death within 5 years after HTX, significantly more patients in the metoprolol group deceased from transplant failure [ivabradine group: 0 of 50 patients (0.0%) vs. metoprolol group: 9 of 54 patients (16.6%); *P* < 0.01]. There was no significant difference between groups concerning acute rejection, infection/sepsis, malignancy, or thromboembolic event/bleeding (all *P* ≥ 0.05). Causes of death within 5 years after HTX are presented in Table 3.

**Table 3:** Causes of death within 5 years after HTX

Parameter	Ivabradine	Metoprolol	Difference (%)	95% CI	*P* value
	(*n* = 50)	(*n* = 54)			
Transplant failure, *n* (%)	0 (0.0%)	9 (16.6%)	16.6	6.6 to 26.6%	< 0.01*
Acute rejection, *n* (%)	0 (0.0%)	1 (1.9%)	1.9	− 1.7 to 5.5%	0.33
Infection/sepsis, *n* (%)	4 (8.0%)	6 (11.1%)	3.1	− 8.2 to 14.4%	0.59
Malignancy, *n* (%)	1 (2.0%)	0 (0.0%)	2.0	− 1.9 to 5.9%	0.30
Thromboembolic event/bleeding, *n* (%)	0 (0.0%)	1 (1.9%)	1.9	− 1.7 to 5.5%	0.33
All causes, *n* (%)	5 (10.0%)	17 (31.5%)	21.5	6.6 to 36.4%	< 0.01*

*CI* confidence interval, *HTX* heart transplantation, *n* = number

*Statistically significant (*P* < 0.05)

Multivariate analysis revealed a four-fold decreased risk of death within 5 years after HTX in patients with post-transplant use of ivabradine (HR 0.25, CI 0.09–0.70; *P* < 0.01), whereas the other variables (recipient age, donor age, transplant sex mismatch, and ischemic time) showed no significant effects on post-transplant mortality. Multivariate analysis for mortality within 5 years after HTX is shown in Table 4.

**Table 4:** Multivariate analysis for mortality within 5 years after HTX

Variable	Hazard ratio	95% CI	*P* value
Administration of ivabradine after HTX (in total)	0.25	0.09–0.70	< 0.01*
Recipient age (years)	1.01	0.96–1.05	0.80
Donor age (years)	1.00	0.96–1.03	0.88
Transplant sex mismatch (in total)	1.30	0.55–3.10	0.55
Ischemic time (min)	1.00	0.99–1.01	0.89

*CI* confidence interval, *HTX* heart transplantation

*Statistically significant (*P* < 0.05)

### Echocardiographic features after heart transplantation

Assessment of echocardiographic features showed a statistically significant reduction of LV mass (*P* < 0.01) and LV mass index (*P* < 0.01) towards normal values in the ivabradine group 5 years after HTX, whereas no such effect over time was found in the metoprolol group in terms of LV mass (*P* = 0.84) or LV mass index (*P* = 0.87).

Within 5 years after HTX, the ivabradine group had no statistically significant change in LVEF (*P* = 0.93) or MAPSE (*P* = 0.70), while the metoprolol group had a slight but significant decrease in LVEF (*P* < 0.01) and MAPSE (*P* < 0.01).

Analysis of diastolic parameters revealed that patients with metoprolol had a decrease in the *E*/*A* ratio (*P* < 0.01), an increase in the *E*/*e*′ ratio (*P* < 0.01), and a stable DT-E (*P* = 0.43) 5 years after HTX. Patients with ivabradine in contrast showed over time a stable *E*/*A* ratio (*P* = 0.20), a stable *E*/*e*′ ratio (*P* = 0.68), and a decrease in DT-E (*P* < 0.01). Five years after HTX, patients with metoprolol had a broader LA diameter (ivabradine group: 39.4 ± 4.9 mm vs. metoprolol group: 42.2 ± 4.6; *P* < 0.01) and patients with ivabradine showed a lower systolic PA pressure (ivabradine group: 25.8 ± 7.5 mmHg vs. metoprolol group: 30.1 ± 6.0 mmHg; *P* < 0.01). Echocardiographic features after HTX are presented in Table 5.

**Table 5:** Echocardiographic features after HTX

Parameter	Ivabradine	Metoprolol	*P* value
	(*n* = 50)	(*n* = 54)	
LV mass (g), mean ± SD			
At baseline	179.5 ± 42.4	178.8 ± 39.6	0.93
At 24-month follow-up	155.5 ± 40.8	182.3 ± 55.2	0.01*
At 60-month follow-up	154.8 ± 23.4	177.3 ± 28.4	< 0.01*
* P* value: baseline vs. 24-month follow-up	< 0.01*	0.72	
* P* value: baseline vs. 60-month follow-up	< 0.01*	0.84	
LV mass index (g/m^2^), mean ± SD			
At baseline	96.4 ± 21.8	92.4 ± 20.6	0.34
At 24-month follow-up	82.3 ± 18.6	91.5 ± 19.1	0.02*
At 60-month follow-up	81.7 ± 13.0	93.1 ± 19.2	< 0.01*
* P* value: baseline vs. 24-month follow-up	< 0.01*	0.84	
* P* value: baseline vs. 60-month follow-up	< 0.01*	0.87	
LVEF (%), mean ± SD			
At baseline	61.9 ± 4.0	62.9 ± 4.0	0.22
At 24-month follow-up	62.3 ± 4.9	56.5 ± 7.7	< 0.01*
At 60-month follow-up	61.8 ± 6.0	56.2 ± 5.1	< 0.01*
* P* value: baseline vs. 24-month follow-up	0.66	< 0.01*	
* P* value: baseline vs. 60-month follow-up	0.93	< 0.01*	
MAPSE (mm), mean ± SD			
At baseline	17.4 ± 1.8	17.9 ± 1.6	0.11
At 24-month follow-up	17.9 ± 2.0	15.8 ± 2.3	< 0.01*
At 60-month follow-up	17.6 ± 2.5	15.4 ± 2.8	< 0.01*
* P* value: baseline vs. 24-month follow-up	0.16	< 0.01*	
* P* value: baseline vs. 60-month follow-up	0.70	< 0.01*	
*E*/*A* ratio, mean ± SD			
At baseline	1.5 ± 0.3	1.5 ± 0.4	0.77
At 24-month follow-up	1.6 ± 0.3	1.3 ± 0.6	< 0.01*
At 60-month follow-up	1.6 ± 0.4	1.3 ± 0.4	< 0.01*
* P* value: baseline vs. 24-month follow-up	0.18	0.03*	
* P* value: baseline vs. 60-month follow-up	0.20	< 0.01*	
E/e′ ratio, mean ± SD			
At baseline	7.4 ± 2.7	7.6 ± 2.8	0.75
At 24-month follow-up	7.0 ± 2.8	9.2 ± 4.3	< 0.01*
At 60-month follow-up	7.2 ± 2.5	9.9 ± 3.9	< 0.01*
* P* value: baseline vs. 24-month follow-up	0.48	0.04*	
* P* value: baseline vs. 60-month follow-up	0.68	< 0.01*	
DT-E (ms), mean ± SD			
At baseline	211.5 ± 23.1	210.4 ± 25.8	0.81
At 24-month follow-up	182.3 ± 23.1	206.3 ± 35.1	< 0.01*
At 60-month follow-up	180.4 ± 20.6	206.1 ± 24.9	< 0.01*
* P* value: baseline vs. 24-month follow-up	< 0.01*	0.53	
* P* value: baseline vs. 60-month follow-up	< 0.01*	0.43	
LA diameter (mm), mean ± SD			
At baseline	38.4 ± 5.9	39.2 ± 5.3	0.46
At 24-month follow-up	38.1 ± 4.9	40.4 ± 5.0	0.03*
At 60-month follow-up	39.4 ± 4.9	42.2 ± 4.6	0.01*
* P* value: baseline vs. 24-month follow-up	0.80	0.26	
* P* value: baseline vs. 60-month follow-up	0.34	< 0.01*	
Systolic PA pressure (mmHg), mean ± SD			
At baseline	30.2 ± 7.6	30.6 ± 7.7	0.79
At 24-month follow-up	26.2 ± 6.8	30.1 ± 7.4	0.01*
At 60-month follow-up	25.8 ± 7.5	30.1 ± 6.0	< 0.01*
* P* value: baseline vs. 24-month follow-up	< 0.01*	0.75	
* P* value: baseline vs. 60-month follow-up	< 0.01*	0.73	

*DT-E* deceleration time (DT) of the early diastolic mitral inflow peak (E), *E*/*A* early diastolic mitral inflow peak velocity (*E*) to late diastolic mitral inflow peak velocity (*A*) ratio, *E/e′* early diastolic mitral inflow peak velocity (*E*) to early diastolic mitral annular velocity (*e′*) ratio, *HTX* heart transplantation, *LA* left atrial, *LV* left ventricular, *LVEF* left ventricular ejection fraction, *MAPSE* mitral annular plane systolic excursion, *PA* pulmonary artery, *SD* standard deviation

*Statistically significant (*P* < 0.05)

### Cardiac catheterization data and cardiac biomarkers after heart transplantation

Cardiac catheterization data showed no statistically significant differences between patients with ivabradine or metoprolol in coronary artery disease, coronary stenting or high-sensitivity cardiac troponin T (all *P* ≥ 0.05). Patients with ivabradine had a significantly lower left ventricular end-diastolic pressure (LVEDP) at 24-month follow-up (ivabradine group: 12.7 ± 3.2 mmHg vs. metoprolol group: 17.0 ± 3.1 mmHg; *P* < 0.01) and at 60-month follow-up (ivabradine group: 12.0 ± 3.7 mmHg vs. metoprolol group: 17.1 ± 2.6 mmHg; *P* < 0.01) as well as a significantly lower N-terminal prohormone of brain natriuretic peptide (NT-proBNP) at 24-month follow-up (ivabradine group: 798.2 ± 1022.8 pg/ml vs. metoprolol group: 1407.5 ± 1290.9 pg/ml; *P* = 0.01) and at 60-month follow-up (ivabradine group: 525.4 ± 555.4 pg/ml vs. metoprolol group: 1038.9 ± 865.0 pg/ml; *P* < 0.01). Cardiac catheterization data and cardiac biomarkers after HTX are given in Table 6.

**Table 6:** Cardiac catheterization data and cardiac biomarkers after HTX

Parameter	Ivabradine	Metoprolol	*P* value
	(*n* = 50)	(*n* = 54)	
Coronary artery disease (stenosis ≥ 50%), *n* (%)			
At baseline	3 (6.0%)	2 (3.7%)	0.58
At 24-month follow-up	11 (22.0%)	12 (22.2%)	0.98
At 60-month follow-up	13 (26.0%)	13 (24.1%)	0.82
* P* value: baseline vs. 24-month follow-up	0.02*	< 0.01*	
* P* value: baseline vs. 60-month follow-up	< 0.01*	< 0.01*	
Coronary stenting, *n* (%)			
At baseline	0 (0.0%)	0 (0.0%)	n.a.
At 24-month follow-up	4 (8.0%)	4 (7.4%)	0.91
At 60-month follow-up	5 (10.0%)	6 (11.1%)	0.85
* P* value: baseline vs. 24-month follow-up	0.04*	0.04*	
* P* value: baseline vs. 60-month follow-up	0.02*	0.01*	
LVEDP (mmHg), mean ± SD			
At baseline	15.5 ± 2.8	15.2 ± 2.1	0.58
At 24-month follow-up	12.7 ± 3.2	17.0 ± 3.1	< 0.01*
At 60-month follow-up	12.0 ± 3.7	17.1 ± 2.6	< 0.01*
* P* value: baseline vs. 24-month follow-up	< 0.01*	< 0.01*	
* P* value: baseline vs. 60-month follow-up	< 0.01*	< 0.01*	
High-sensitivity cardiac troponin T (pg/ml), mean ± SD			
At baseline	167.7 ± 69.8	166.9 ± 81.2	0.96
At 24-month follow-up	18.4 ± 11.9	21.4 ± 12.5	0.25
At 60-month follow-up	14.4 ± 11.6	18.3 ± 10.9	0.13
* P* value: baseline vs. 24-month follow-up	< 0.01*	< 0.01*	
* P* value: baseline vs. 60-month follow-up	< 0.01*	< 0.01*	
NT-proBNP (pg/ml), mean ± SD			
At baseline	3826.3 ± 2002.8	3858.9 ± 1756.3	0.93
At 24-month follow-up	798.2 ± 1022.8	1407.5 ± 1290.9	0.01*
At 60-month follow-up	525.4 ± 555.4	1038.9 ± 865.0	< 0.01*
* P* value: baseline vs. 24-month follow-up	< 0.01*	< 0.01*	
* P* value: baseline vs. 60-month follow-up	< 0.01*	< 0.01*	

*HTX* heart transplantation, *LVEDP* left ventricular end-diastolic pressure, *n* number, *n.a.* not applicable, *NT-proBNP* N-terminal prohormone of brain natriuretic peptide, *SD* standard deviation

*Statistically significant (*P* < 0.05)

## Discussion

### Long-term management of resting heart rate

Our investigation is the first study on 5-year results of heart rate control with ivabradine or metoprolol in patients after HTX. We found a significantly better long-term heart rate reduction in patients with ivabradine than in patients with metoprolol. Heart rate reduction with ivabradine was associated with a normalization of LV mass, a lower LVEDP, a lower NT-proBNP level, and a lower 5-year mortality after HTX.

In this study, patients with ivabradine or metoprolol showed a comparable mean resting heart rate at baseline visit after HTX. Already at first follow-up, the ivabradine group did not only have a significantly lower heart rate compared to baseline but also to the metoprolol group. This prompt and significant decline of heart rate shows the efficacy of ivabradine in patients after HTX. Previous studies required more time to achieve statistical significance in heart rate reduction between groups which might be a result of smaller sample size [9–14]. Patients with ivabradine sustained a significantly lower resting heart rate than patients with metoprolol over the entire 5-year period. This is in line with two former studies investigating short-term effects of heart rate control with ivabradine or metoprolol in patients after HTX [9, 14].

For a better understanding of the superior resting heart rate reduction with ivabradine compared to metoprolol in patients after HTX, it is essential to take a closer look at the physiological aspects of the cardiac allograft and the mechanism of action of both drugs. In non-transplant patients, the resting heart rate is mainly controlled by the autonomic nervous system. The autonomic nervous system is divided into the sympathetic and parasympathetic nervous system which both innervate the sinoatrial node. Parasympathetic activity lowers the heart rate and sympathetic activity increases the heart rate. At rest, the parasympathetic nervous system predominates and causes a decline of the spontaneous heart rate of the sinoatrial node from about 100–110 bpm down to a resting heart rate of 60–80 bpm [35–37].

As a consequence of surgical denervation after HTX, chronotropic control of the autonomic nervous system—especially the vagal heart rate reduction of the intrinsic sinoatrial node activity—is rescinded resulting in an elevated resting heart rate [38]. There is currently no medication or surgical procedure available to restore cardiac innervation, thus heart rate reduction is limited to pharmacological treatment. Ivabradine is a specific and selective inhibitor of HCN channels which are responsible for the generation of the “pacemaker current” I_f_ in the sinoatrial node. Ivabradine therefore causes a direct reduction of the heart rate independently of the autonomic nervous system [20–24]. In contrast, metoprolol is a beta blocker which indirectly lowers the heart rate by inhibiting the epinephrine- and norepinephrine-mediated sympathetic actions on beta-adrenergic receptors [38]. The use of beta blockers in patients after HTX is hence less effective as the chronotropic control of the autonomic nervous system is rescinded [9, 14].

Differences in dosage between patients with ivabradine or metoprolol may have influenced heart rate reduction. Mean daily ivabradine dose (2 × 5.0 mg) and metoprolol dose (2 × 50 mg or 1 × 100 mg) met the clinical standard at baseline visit. Patients in both groups received a similar dose increase over time but maximum daily dose of ivabradine (2 × 7.5 mg) or metoprolol (2 × 100 mg or 1 × 200 mg) was not administered in all patients often due to patient reports of temporary asymptomatic bradycardia (heart rate < 60 bpm) during self-measurements [10, 11, 14].

### Impact on blood pressure and side effects

In this study, patients with ivabradine or metoprolol had a comparable systolic and diastolic blood pressure at baseline. Systolic and diastolic blood pressure in patients with ivabradine was unaltered over time and we found no significant difference between patients with ivabradine or metoprolol regarding systolic and diastolic blood pressure at 2-year or 5-year follow-up. These findings are in accordance with former studies which found no significant differences in systolic and diastolic blood pressure as ivabradine is a specific and selective inhibitor of HCN channels reducing heart rate without affecting blood pressure, atrioventricular conduction, or inotropy [9–14, 20].

Although ivabradine has been shown to be safe and effective in heart rate reduction in patients after HTX [9–14], the use of ivabradine is still off-label in these patients [34]. In this study, patients with ivabradine had a significantly lower percentage of symptomatic bradycardia, dizziness, fatigue, and sexual dysfunction in comparison to patients with metoprolol. A specific side effect of ivabradine is the perception of luminous phenomena (phosphenes) as a result of inhibition of a structurally similar retinal channel. However, these phosphenes are reported to appear about 40 days after treatment begins, to be temporary, and not to negatively affect patients’ daily lives [9–14, 20].

### Survival after heart transplantation

Regulation of resting heart rate is essential in patients after HTX as a rapid heartbeat has been associated with increased post-transplant mortality [14, 15]. Ivabradine has been shown to be safe and effective in the treatment of elevated heart rates in patients after HTX [9–14]. However, results are limited to short and mid-term findings and long-term data are not available [9–14]. We found a significantly better 5-year survival in patients after HTX with ivabradine along with a lower number of deaths due to transplant failure. Extensive analyses of demographics, clinical characteristics, post-transplant medication and immunosuppressive drug therapy between both groups revealed no statistically significant differences potentially affecting mortality after HTX. Multivariate analysis further showed a four-time lower mortality risk in patients with ivabradine after HTX indicating beneficial effects on long-term survival for patients with ivabradine after HTX.

### Post-transplant effects of ivabradine

Several mechanisms seem to be involved in the cardioprotective effects of ivabradine [25–28]. Heart rate reduction causes a prolongation of diastolic time improving coronary blood flow, ventricular filling and diastolic function [39, 40]. Improvement of diastolic function may be explained by increased sarcoplasmic reticulum calcium up-take and ATPase (SERCA) activity [40]. In our study, patients with ivabradine had a significantly higher *E*/*A* ratio and a significantly lower *E*/*e*′ ratio which is in line with former reports [25, 40]. Moreover, ivabradine can improve myocardial structure and systolic function by modification of cardiac myocytes and the extracellular matrix [26, 27]. We found a better LVEF in patients with ivabradine along with a significant reduction of LV mass and LV mass index towards normal values. Cardiac remodeling and attenuation of cardiomyocyte hypertrophy by ivabradine may further improve LV function by alleviating hypoxia and lowering myocardial oxygen consumption [40].

Every single heartbeat consumes energy and causes mechanical stress on the endothelial cells [41]. Elevated heart rates induce increased pulsatile stretch which can lead to endothelial dysfunction, inflammation, degradation, and microvascular coronary disease [25, 26, 40, 41]. Long-term use of ivabradine has been described to induce angiogenesis augmenting microvascular coronary perfusion. Patients with ivabradine or metoprolol showed no significant difference in coronary artery disease or coronary stenting in this study. However, patients with ivabradine showed a significantly lower LVEDP and NT-proBNP indicating a potential positive effect of ivabradine on the microvascular level. The use of ivabradine has been demonstrated to inhibit the accumulation of reactive oxygen species which might be beneficial, as subclinical myocardial ischemia and oxidative stress are common in diastolic dysfunction [42, 43]. Moreover, inhibition of HCN channels can decrease cardiac mitochondrial oxygen consumption [44]. In contrast to ivabradine, the use beta blocker has been linked to α-adrenergic coronary vasoconstriction. This raises the question, whether a switch from metoprolol to ivabradine may improve microvascular coronary blood flow [25]. Improvement of microvascular coronary perfusion, reduction of oxygen consumption and reactive oxygen species, and protective vascular effects are pivotal, as about one-third of patients after HTX develop cardiac allograft vasculopathy by 5 years post-transplant due to coronary inflammation, endothelial dysfunction and vascular fibroproliferation [45].

All the above-mentioned examples show that the use of ivabradine in patients after HTX does not only offer an excellent therapy for heart rate reduction but also triggers changes on different levels including improvement of systolic and diastolic function, normalization of cardiomyocyte hypertrophy and augmentation of microvascular coronary perfusion by induced angiogenesis [25–27, 40, 41]. Therefore, in the light of growing organ shortage over the last years, [46, 47] our findings are of great clinical value as we could demonstrate in this study that ivabradine is not simply a suitable alternative in case of beta blocker intolerance but provides additional beneficial effects. As a result of this study and previous investigations on ivabradine in patients after HTX, ivabradine has now become standard for heart rate control in patients after HTX at Heidelberg Heart Center.

### Study limitations

Our study results were based on a single-center registry (Heidelberg HTX Registry) with 104 adult patients receiving HTX at Heidelberg Heart Center. Hereof, 50 patients had ivabradine and 54 had metoprolol succinate after HTX. Findings should be interpreted with caution as the non-randomized study design carries certain limitations and may be subject to unmeasured confounders. However, an advantage of this single-center study is the standardized center-specific pre-, peri-, and post-transplant course of treatment and follow-up of patients after HTX. Regarding the rather small number of patients, our study is the first and thereby largest study analyzing 5-year results of heart rate control with ivabradine or metoprolol succinate in patients after HTX. It therefore provides important and clinically relevant information [14].

Devereux formula was used to calculate LV mass which carries the limitation of two-dimensional assessment. As a matter of fact, no more accurate assessment of LV mass by three-dimensional echocardiography has yet been established as a standard method of clinical measurement. We did not perform a randomization of patients regarding the use of ivabradine or metoprolol as physician practice and patient preference influenced the prescription reflecting real-world data. However, we could not detect significant differences between groups in terms of demographics or concurrent drugs reducing the likelihood of selection bias. Regarding the use of ivabradine or metoprolol succinate over 5 years, patients after HTX showed a very high rate of medication adherence as this is crucial for their survival. In addition, patients were routinely asked about their medication adherence at each follow-up and change of medication was standardly performed only after consultation [14].

Importantly, our results should be considered as hypothesis-generating, especially in terms of post-transplant survival as multiple factors may affect survival after HTX. It is further unknown, whether these effects are attributed to differences between ivabradine and metoprolol or ivabradine and beta blockers in general. To confirm our findings, large, prospective randomized controlled multi-center trials are required to investigate the effects of ivabradine and metoprolol on post-transplant outcomes.

## Conclusion

We performed the first study on 5-year results of heart rate control with ivabradine or metoprolol in patients after HTX. At 5-year follow-up, patients with ivabradine had a significantly lower heart rate (*P* < 0.01), a lower left ventricular end-diastolic pressure (*P* < 0.01), and a lower NT-proBNP level (*P* < 0.01) in comparison to the baseline visit or patients with metoprolol. Moreover, patients with ivabradine showed a significantly better 5-year post-transplant survival in contrast to patients with metoprolol (*P* < 0.01). In summary, a specific and selective long-term modulation of cardiac chronotropic function with ivabradine in patients after HTX was associated with a pronounced heart rate reduction, an improved left ventricular diastolic function and an increased 5-year survival after HTX.
