# Correction to: Five-year results of heart rate control with ivabradine or metoprolol succinate in patients after heart transplantation

**DOI:** 10.1007/s00392-021-01948-2

**Published:** 2021-10-26

**Authors:** Rasmus Rivinius, Matthias Helmschrott, Ann-Kathrin Rahm, Fabrice F. Darche, Dierk Thomas, Tom Bruckner, Andreas O. Doesch, Hugo A. Katus, Philipp Ehlermann

**Affiliations:** 1 Department of Cardiology, Angiology and Pneumology, Heidelberg University Hospital, Im Neuenheimer Feld 410, 69120 Heidelberg, Germany; 2 Heidelberg Center for Heart Rhythm Disorders (HCR), Heidelberg University Hospital, Heidelberg, Germany; 3 German Center for Cardiovascular Research (DZHK), Partner Site Heidelberg/Mannheim, Heidelberg, Germany; 4 Institute for Medical Biometry and Informatics, University of Heidelberg, Heidelberg, Germany; 5 Department of Pneumology and Oncology, Asklepios Hospital, Bad Salzungen, Germany

## Correction to: Clinical Research in Cardiology 10.1007/s00392-020-01692-z

The article Five-year results of heart rate control with ivabradine or metoprolol succinate in patients after heart transplantation, written by Rasmus Rivinius, Matthias Helmschrott, Ann-Kathrin Rahm, Fabrice F. Darche, Dierk Thomas, Tom Bruckner, Andreas O. Doesch, Hugo A. Katus, Philipp Ehlermann, was originally published electronically on the publisher’s internet portal on 22 June 2020 without open access. With the author(s)’ decision to opt for Open Choice the copyright of the article changed on 27 May 2021 to © The Author(s) 2020 and the article is forthwith distributed under a Creative Commons Attribution 4.0 International License, which permits use, sharing, adaptation, distribution and reproduction in any medium or format, as long as you give appropriate credit to the original author(s) and the source, provide a link to the Creative Commons licence, and indicate if changes were made.

The images or other third party material in this article are included in the article’s Creative Commons licence, unless indicated otherwise in a credit line to the material. If material is not included in the article’s Creative Commons licence and your intended use is not permitted by statutory regulation or exceeds the permitted use, you will need to obtain permission directly from the copyright holder.

To view a copy of this licence, visit http://creativecommons.org/licenses/by/4.0/.

